# Molecular evolution of human coronavirus-NL63, -229E, -HKU1 and -OC43 in hospitalized children in China

**DOI:** 10.3389/fmicb.2022.1023847

**Published:** 2022-11-02

**Authors:** Nan Shao, Chi Zhang, Jie Dong, Lilian Sun, Xiangpeng Chen, Zhengde Xie, Baoping Xu, Shuhua An, Ting Zhang, Fan Yang

**Affiliations:** ^1^NHC Key Laboratory of Systems Biology of Pathogens, Institute of Pathogen Biology, Chinese Academy of Medical Sciences & Peking Union Medical College, Beijing, China; ^2^Key Laboratory of Respiratory Disease Pathogenomics, Chinese Academy of Medical Sciences & Peking Union Medical College, Beijing, China; ^3^Beijing Key Laboratory of Pediatric Respiratory Infection Diseases, Key Laboratory of Major Diseases in Children, Ministry of Education, National Clinical Research Center for Respiratory Diseases, Research Unit of Critical Infection in Children, Chinese Academy of Medical Sciences, 2019RU016, Laboratory of Infection and Virology, Beijing Pediatric Research Institute, Beijing Children’s Hospital, Capital Medical University, National Center for Children’s Health, Beijing, China; ^4^National Clinical Research Center for Respiratory Diseases, Research Unit of Critical Infection in Children, Chinese Academy of Medical Sciences, 2019RU016, Respiratory department, Beijing Children’s Hospital, Capital Medical University, National Center for Children’s Health, Beijing, China; ^5^Hebei Province Children’s Hospital, Shijiazhuang, Hebei, China

**Keywords:** human coronavirus, spike gene, phylogenetic analysis, recombination analysis, *N*-glycosylation site, amino acid site

## Abstract

Human coronaviruses (HCoVs) HCoV-NL63, HCoV-229E, HCoV-HKU1 and HCoV-OC43 have been circulated in the human population worldwide, and they are associated with a broad range of respiratory diseases with varying severity. However, there are neither effective therapeutic drugs nor licensed vaccines available for the treatment and prevention of infections by the four HCoVs. In this study, we collected nasopharyngeal aspirates of children hospitalized for respiratory tract infection in China during 2014–2018 and conducted next-generation sequencing. Sequences of four HCoVs were then selected for an in-depth analysis. Genome sequences of 2 HCoV-NL63, 8 HCoV-229E, 2 HCoV-HKU1, and 6 HCoV-OC43 were obtained. Based on the full-length *S* gene, a strong temporal signal was found in HCoV-229E and the molecular evolutionary rate was 6 × 10^−4^ substitutions/site/year. Based on the maximum-likelihood (ML) phylogenetic tree of complete *S* gene, we designated H78 as a new sub-genotype C2 of HCoV-HKU1, and the obtained P43 sequence was grouped into the reported novel genotype K of HCoV-OC43 circulating in Guangzhou, China. Based on the complete genome, potential recombination events were found to occur as two phenomena, namely intraspecies and interspecies. Moreover, we observed two amino acid substitutions in the S1 subunit of obtained HCoV-NL63 (G534V) and HCoV-HKU1 (H512R), while residues 534 and 512 are important for the binding of angiotensin-converting enzyme 2 and neutralizing antibodies, respectively. Our findings might provide a clue for the molecular evolution of the four HCoVs and help in the early diagnosis, treatment and prevention of broad-spectrum HCoV infection.

## Introduction

Coronaviruses (CoVs) belong to the order Nidovirales, family Coronaviridae and subfamily Coronavirinae. Coronavirinae is further divided into four genera—*Alphacoronavirus* (α-CoV), *Betacoronavirus* (β-CoV), *Gammacoronavirus* (γ-CoV) and *Deltacoronavirus* (δ-CoV; Fung and Liu, 2019; Chen et al., 2020). Among these, the β-CoVs are subdivided into four lineages, namely A, B, C and D. CoVs are spherical, enveloped, non-segmented, linear and positive-sense, single-stranded RNA viruses. With a capped and polyadenylated viral genome of approximately 26–32 kb, they are the largest known RNA viruses to date. All CoVs have a common characteristic of genome organization. In the 5′ end, two-thirds of the genome consists of two overlapping open reading frames (ORF1a and ORF1b), encoding the non-structural proteins pivotal to RNA replication. In the 3′ end, one-third of the genome possesses four structural proteins (S, E, M and N; Lim et al., 2016).

Since the emergence of coronavirus disease 2019 at the end of 2019, seven HCoVs have been found to infect humans, namely HCoV-229E, HCoV-NL63, HCoV-HKU1, HCoV-OC43, severe acute respiratory syndrome coronavirus (SARS-CoV), Middle East respiratory syndrome coronavirus (MERS-CoV) and SARS-CoV-2 (Dhama et al., 2020; Zhu et al., 2020). Among them, HCoV-229E and HCoV-NL63 are members of α-CoVs, which were first identified in 1962 in the United States and 2004 in the Netherlands, respectively (Hamre and Procknow, 1966; Su et al., 2016). The other five HCoVs have been classified as β-CoVs with different lineages, and they emerged in different years. Of these, HCoV-OC43 and HCoV-HKU1 belong to lineage A and were first characterized in 1967 in France and 2005 in China, respectively (Su et al., 2016).

The seven HCoVs are known to circulate globally and are associated with a broad range of diseases with varying severity (Su et al., 2016). Among these, the four HCoVs (HCoV-NL63, HCoV-229E, HCoV-HKU1 and HCoV-OC43) normally cause asymptomatic, self-limited and mild respiratory tract symptoms (Zumla et al., 2016). Occasionally, four HCoVs can also result in moderate to even severe respiratory infections, especially for susceptible infants, young children, elderly people, immunocompromised individuals and people with underlying diseases or comorbidities (Hasoksuz et al., 2020; Rajapakse and Dixit, 2021; Veiga et al., 2021). Globally, HCoV-NL63, HCoV-229E, HCoV-HKU1, and HCoV-OC43 infections account for up to 30% of human respiratory tract illnesses (Lim et al., 2016; Fung and Liu, 2019). In addition, the above four HCoVs can cause either single infection or co-infection with other respiratory viruses (Zeng et al., 2018). Moreover, a previous study reported that HCoV-OC43 and HCoV-NL63 infections could trigger immune responses against the subsequent HCoV-HKU1 and HCoV-229E infections, respectively (Dijkman et al., 2012). However, there are neither effective therapeutic drugs nor licensed vaccines against the four HCoVs thus far. Hence, socio–economic effects of the four HCoVs require more attention.

Recently, two novel lineages (J and K) of HCoV-OC43 have been detected in Guangzhou, China (Zhang et al., 2022). Besides, a new sub-genotype C3 of HCoV-NL63 was also discovered in Guangzhou, China, meanwhile, a mutation (I507 L) in receptor-binding domain (RBD) associated with enhanced viral entry was identified in this new sub-genotype (Wang et al., 2020). Notably, HCoV-NL63 shares the same cell receptor, angiotensin-converting enzyme 2 (ACE2), as SARS-CoV and SARS-CoV-2, in contrast to HCoV-229E, HCoV-OC43 and HCoV-HKU1 (Lim et al., 2016). Moreover, potential recombination events of the four HCoVs have been reported (Woo et al., 2006; Lau et al., 2011; Su et al., 2016; Tao et al., 2017; Pollett et al., 2021). Therefore, the molecular characteristics of the four HCoVs in the north of China attracted our attention. However, the number of the publicly-available complete genomes of the four HCoVs, especially those of HCoV-NL63, HCoV-229E and HCoV-HKU1, in the NCBI database is insufficient.

More sequence information is beneficial for in-depth analysis to elucidate the molecular evolutionary characteristic of HCoV-NL63, −229E, -HKU1, and -OC43, such as the nucleotide identity, phylogenetics, potential recombination event, N-glycosylation site, and major amino acid substitution site. These characteristics could bring us more thorough understanding of HCoVs. Notably, *S* gene of HCoVs have been reported important for the receptor-binding and membrane-fusion, phylogenetics and potential recombination events, which contribute to further analyses of the genotype shift and circulation (Ren et al., 2015; Zhang et al., 2015; Xia et al., 2019; Eguia et al., 2021). Besides, N-glycosylation of *S* gene is considered to be important for viral antigenicity and some biological activities in the β-CoV of SARS-CoV-2 (Wang et al., 2021). Therefore, in order to obtain more genome sequence information of the four HCoVs, we collected the nasopharyngeal aspirates of hospitalized children with respiratory tract infection and performed next-generation sequencing (NGS) in this study. Our study might provide new insights into the diagnosis, prevention, treatment, vaccine development and control strategies of virus-mediated infections.

## Materials and methods

### Sample collection and nucleic acid extraction

Clinical samples were collected from two hospitals in China during 2014–2018. Viral particles were enriched in all samples, as described previously (Liu et al., 2020). Viral nucleic acids were extracted using the QIAamp 96 Virus QIAcube HT Kit and the QIAxtractor instrument (Qagien, Germany).

### Double-stranded cDNA synthesis and PCR amplification

The viral nucleic acid was converted from first-strand complementary DNA (cDNA) to double-stranded cDNA (ds cDNA) using the same procedure as described previously (Shao et al., 2021). PCR amplification was conducted with a final volume of 50 μl, consisting of 4.7 μl ds cDNA templates, 2.5 μl primer K (20 pM), 25 μl Premix Taq (Takara, Japan), and 17.8 μl nuclease-free water. The amplification reaction was performed using the following thermal cycling program: 94°C for 5 min, followed by 30 cycles at 94°C for 30 s, 50°C for 30 s and 72°C for 2 min, and finally at 72°C for 10 min. The PCR amplification products were purified using QIAquick PCR Purification Kit and then quantified using Qubit^™^ 3.0 Fluorometer and Qubit^™^ dsDNA High Sensitivity Assay Kit (Invitrogen, United States). Subsequently, each sample was diluted to an equal concentration (300 pg./μl) and prepared for NGS sequencing.

### NGS sequencing and genome assembly

Libraries were constructed using Nextera^®^ XT DNA Library Prep Kit (Illumina, United States) in accordance with the manufacturer’s instructions. AMPure XP beads (Beckman Coulter, United States) were used to perform size selection and purification of fragments for the libraries, and the size of fragments for each library was assessed using the Agilent 2,100 Bioanalyzer (Agilent, United States). The pooled libraries were sequenced using the Illumina Hiseq X Ten PE150 platform at Annoroad Gene Technology Co., Ltd., (Beijing, China).

Adapter-contaminated reads, low-quality reads and Ns reads (>5%) were removed from the raw reads. Valid reads from high-throughput sequencing were assembled and manually edited using the Geneious Prime 2020^1^ and SeqMan program (DNASTAR, United States). Read mapping was performed using the respective reference sequences for HCoV-NL63 (accession numbers: NC_005831 and MK334043), HCoV-229E (accession numbers: NC_002645 and MN369046), HCoV-HKU1 (accession numbers: NC_006577 and AY884001) and HCoV-OC43 (accession numbers: NC_006213 and MN306041). The remaining gaps in the genome were filled using targeted PCR amplification and sequencing.

### Identity analysis

Full genome sequences of HCoV-NL63, HCoV-229E, HCoV-HKU1 and HCoV-OC43 were downloaded from the GenBank database on November 20, 2021. Of those, incomplete genome sequences were excluded. All obtained sequences and the corresponding retrieved complete genome sequences were aligned using the tools Clustal Omega and MAFF.^2^ Alignment sequences of four HCoVs were trimmed using BioEdit software. Identity was analysed using the DNASTAR MegAlign software.

### Molecular phylogenetic analysis

The *S* gene sequences of HCoV-NL63, HCoV-229E, HCoV-HKU1 and HCoV-OC43 available at NCBI, were retrieved on November 20, 2021. Among them, we excluded the sequences of laboratory-adapted strains, cloned strains and strains with unclear collection years and country. Furthermore, sequences that encompassed incomplete coding sequences and degenerate bases were omitted. For identical sequences from the same strain, only one sequence was considered.

To explore the temporal signal of the complete *S* gene for the four HCoVs datasets, an individual maximum likelihood phylogenetic tree was constructed using ModelFinder implemented in the W-IQ-TREE Web server (Trifinopoulos et al., 2016). Phylogenetic analysis was performed using a non-molecular clock and best-fit model with 1,000 replicates. TempEst v1.5.3 was used to visualize the root-to-tip plot of time-stamped sequences based on the regression analysis.

The complete *S* gene sequences of the HCoV dataset with temporal signal was used to conduct the time-scaled phylogenetic tree inferences using the Bayesian Markov Chain Monte Carlo (MCMC) approach implemented in BEAST v.1.10.4.^3^ The evolutionary model consisted of GTR + G + I, four gamma categories and three partitioned codon positions. Here, the appropriate nucleotide substitution model was selected according to the Bayesian scores recommended by ModelFinder. Besides, four combinations of two molecular clock models (strict clock and uncorrelated relaxed clock with log-normal distribution (UCLN)) and two coalescent tree prior models (constant-size and exponential growth with growth rate) were tested using the path-sampling (PS)/stepping-stone sampling (SS) method (Baele et al., 2012). Marginal likelihood estimation settings in PS/SS were specified as 100 path steps and one million chain lengths, and the power posteriors were evenly spaced quantiles of a beta (0.3, 1.0) distribution (Xie et al., 2011). The prior distribution of clock rate was set the continuous-time Markov chain rate reference and the length of the MCMC chain was set to 50 million, sampled every 1,000 steps. The XML files created in BEAUti v1.10.4 were run in BEAST. The produced log files were loaded in Tracer v1.10.4 and all parameter estimations with an effective sample size (ESS) over 200 indicated sufficient convergence. Next, a maximum clade credibility (MCC) tree with 10% burn-in was built using the TreeAnnotator program in the BEAST package. The final step was to visualize the accompanying summary information of the MCC tree in FigTree v1.10.4.

The complete *S* gene sequences of the HCoV dataset lacking the temporal signal was used to construct maximum-likelihood (ML) phylogenetic tree using MEGA v6.0 with 1,000 bootstrap replicates. Of these, the best-fit substitution model of TN93 + G + I was recommended by the model function in the program MEGA v6.0 (Tamura et al., 2013) with 1,000 bootstrap replicates. Besides, the evolutionary divergence among different groups (involving genotype and sub-genotype) of the *S* gene sequences of the HCoV dataset was estimated using Maximum Composite Likelihood model with 1,000 bootstrap replicates in the program MEGA v6.0.

### Recombination analysis

Based on the whole genomes, potential recombination detection was implemented in Recombination Detection Program of RDP5 (Martin et al., 2021). Potential recombination signal identified by RDP5 was subjected to similarity plot and bootscan analysis using SimPlot version 3.5.1.^4^ The sliding window size of 500 bp and a moving step size of 50 bp.

### N-glycosylation site analysis

Considering the importance of *N*-glycosylation in viral antigenicity and some biological activities, we examined *N*-glycosylation sites located in the amino acid (aa) sequence of the S protein of the four obtained HCoVs using NetNGlyc 1.0 server (Gupta and Brunak, 2002). Only when the threshold score was higher than 0.5 and no proline was present at the position of Xaa (Asn-Xaa-Ser/Thr sequons), the N-glycosylation position was considered to be detected.

### Amino acid site analysis

Amino acid substitutions in the S protein were located relative to the prototype strain NC_00583. Homology modelling analysis was performed according to the template of published model in Protein Data Bank (PDB) *via* SWISS-MODEL (Waterhouse et al., 2018). The positions of aa substitution and N-linked glycosylation were also labelled on the three-dimensional structure model of crystal structure. Finally, the S-glycoprotein trimer structures of the obtained HCoV-NL63 were visualized and analysed using PyMOL (Schrödinger). Additionally, the conserved residues of the S glycoproteins were clarified using ESPript 3 (Gouet et al., 2003).

## Results

### Samples and sequences

In this study, we focused on the 18 nasopharyngeal aspirates of children with HCoV infection, 13 were collected from Beijing and 5 from Hebei, China. The clinical characteristics of the 18 hospitalized paediatric patients are shown in Table 1. All patients were less than 6 years of age: 11 were males and 7 were females. Overall, 18 consensus sequences of HCoV were obtained in this study, including 2 HCoV-NL63, 8 HCoV-229E, 2 HCoV-HKU1 and 6 HCoV-OC43. Of these, 15 HCoV sequences (1 HCoV-NL63, 8 HCoV-229E, 2 HCoV-HKU1 and 4 HCoV-OC43) encompassed the complete genomes or nearly complete genomes missing only short sequences in the 5′ or 3′ end. Three HCoV sequences (1 HCoV-NL63 and 2 HCoV-OC4 3) had base deletions in genes *1ab* (H45, H31 and R125) and *S* (H45 and R125). Besides, the common respiratory viruses that co-infected with the four HCoVs included HRV, RSV, Adv and HPIV3.

**Table 1:** Information of four human coronaviruses identified in this study.

Virus	Accession number	Name	Year	Age	Gender	Sample	City	Genome
NL63	OK073075	H45	2014	0.98	female	Nasopharyngeal aspirates	Hebei	Gap in 1ab (234 bp) and S (72 bp)
NL63	OK073076	RS2	2017	1.00	female	Nasopharyngeal aspirates	Beijing	Complete
229E	OK073077	R2	2018	4.00	female	Nasopharyngeal aspirates	Beijing	Complete
229E	OK073078	R10	2017	3.00	male	Nasopharyngeal aspirates	Beijing	Nearly Complete
229E	OK073079	R22	2018	0.05	male	Nasopharyngeal aspirates	Beijing	Complete
229E	OK073080	R35	2018	2.00	male	Nasopharyngeal aspirates	Beijing	Nearly Complete
229E	OK073081	R39	2018	0.05	female	Nasopharyngeal aspirates	Beijing	Nearly Complete
229E	OK073082	R41	2018	5.00	male	Nasopharyngeal aspirates	Beijing	Complete
229E	OK073083	RS15	2017	0.72	male	Nasopharyngeal aspirates	Beijing	Complete
229E	OK073084	P42	2017	0.50	male	Nasopharyngeal aspirates	Beijing	Complete
HKU1	OK073085	R63	2015	6.00	female	Nasopharyngeal aspirates	Beijing	Complete
HKU1	OK073086	H78	2015	3.00	male	Nasopharyngeal aspirates	Hebei	Complete
OC43	OK073087	H10	2014	0.28	male	Nasopharyngeal aspirates	Hebei	Complete
OC43	OK073088	H31	2014	0.50	female	Nasopharyngeal aspirates	Hebei	Gap in 1ab (5 bp)
OC43	OK073089	H49	2015	0.09	female	Nasopharyngeal aspirates	Hebei	Complete
OC43	OK073090	P43	2017	0.92	male	Nasopharyngeal aspirates	Beijing	Complete
OC43	OK073091	R67	2015	2.00	male	Nasopharyngeal aspirates	Beijing	Complete
OC43	OK073092	R125	2016	0.83	male	Nasopharyngeal aspirates	Beijing	Gap in 1ab (100 bp) and S (24 bp)

### Identity analysis

The nucleotide identities of different fragments of 62 HCoV-NL63, 44 HCoV-229E, 42 HCoV-HKU1, and 193 HCoV-OC43 are shown in Table 2. Specifically, nucleotide identities between the obtained sequences and downloaded sequences were 95.4–100%, 96.1–100%, 84.2–100% and 89.7–100%, respectively. Among the obtained sequences, 2 HCoV-NL63, 8 HCoV-229E, 2 HCoV-HKU1, and 6 HCoV-OC43 shared nucleotide identities of 95.8–99.0%, 99.4–100%, 84.1–96.0% and 90.5–100%, respectively. Obviously, 8 obtained HCoV-229E sequences showed a high level of nucleotide identity. Despite partial base deletions in genes *1ab* and *S*, minor differences in nucleotide identity were observed between the 2 HCoV-NL63 sequences and the corresponding downloaded sequences, as well as the 6 HCoV-OC43 sequences and the corresponding downloaded sequences. Thus, the obtained 18 HCoV sequences were subjected to subsequent analysis.

**Table 2:** Nucleotide identity of four human coronaviruses.

Virus	Nucleotide identity (%)					
	CDS	1ab	S	E	M	N
Alphacoronavirus						
HCoV-NL63	95.8–99.8	95.4–99.8	95.4–99.8	95.4–100	95.4–100	95.4–100
	(96.2)	(95.8)	(96.2)	(98.3)	(98.5)	(99.0)
HCoV-229E	98.1–99.9	98.4–99.9	96.1–99.9	99.1–100	97.9–100	97.4–100
	(99.7–100)	(99.7–100)	(99.8–100)	(100)	(99.4–100)	(99.4–100)
Betacoronavirus						
HCoV-HKU1	93.3–99.3	95.8–99.2	84.2–99.8	85.9–99.6	92.0–100	93.3–100
	(93.5)	(96.0)	(84.1)	(85.9)	(92.1)	(93.4)
HCoV-OC43	97.3–100	97.5–100	94.4–100	89.7–100	94.8–100	91.1–100
	(97.9–99.8)	(98.4–99.8)	(94.6–99.8)	(90.5–100)	(99.0–100)	(98.5–100)

The values in parentheses indicate nucleotide identity between sequences obtained in this study.

### Temporal signal analysis

**Figure 1:**
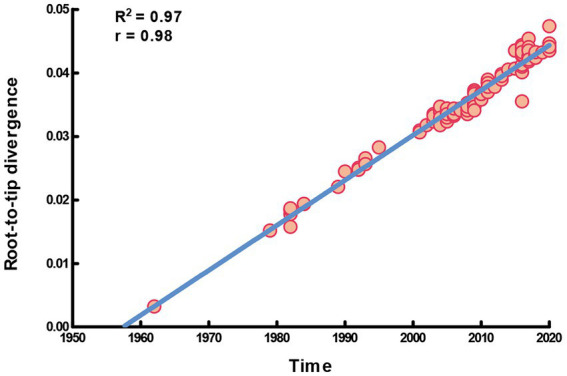
Temporal signal plot of full-length S gene of HCoV-229E. The y-axis and x-axis denoted the regression of root-to-tip divergence against sampling dates.

In this study, the complete *S* gene of four alignments (91 HCoV-NL63, 146 HCoV-229E, 101 HCoV-HKU1 and 142 HCoV-OC43) was used to perform molecular phylogenetic analysis. The root-to-tip regression results in TempEst v1.5.3 showed a relationship between genetic divergence and time and estimated the most recent common ancestor (TMRCA). Apparently, a strong temporal signal (R^2^ = 0.97, correlation coefficient = 0.98) was found in HCoV-229E. The evolutionary rate (i.e., slope value) was approximately 7.09 × 10^−4^ substitutions/site/year, a, and the estimation of TMRCA (i.e., X-intercept value) date was 1957.44 (Figure 1). On the other hand, there was a lack of evidence supporting the temporal signal of HCoV-NL63, HCoV-HKU1 and HCoV-OC43. Therefore, only the dataset of HCoV-229E was performed the time-scaled phylogenetic analysis.

### Molecular phylogenetic analysis

**Figure 2:**
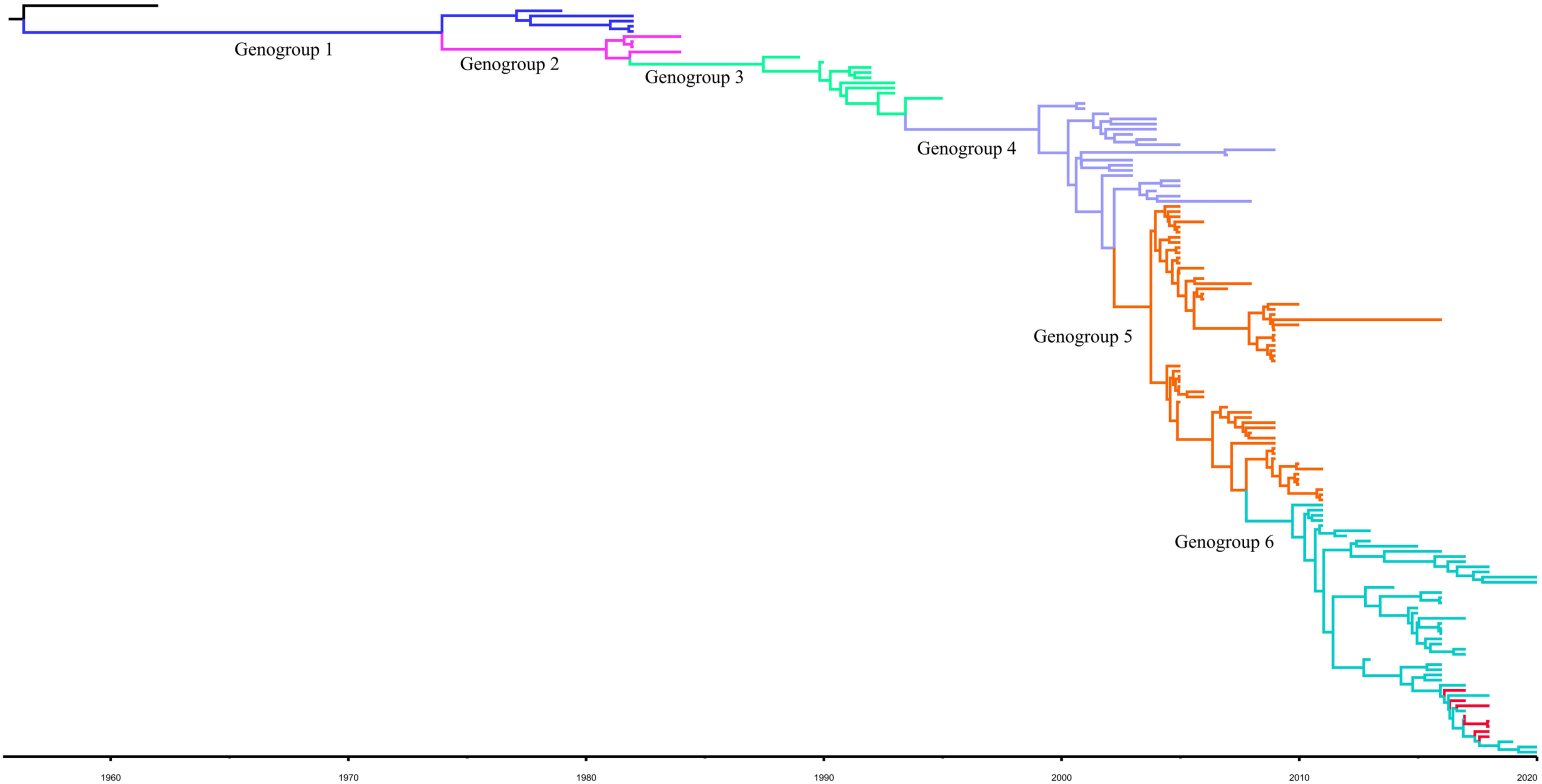
Bayesian MCC tree for the complete S gene of HCoV-229E. Genotype classification is color-coded and sequences obtained in this study are colored red. BEAST v.1.10.4 was used to estimate the most recent common ancestor (tMRCA). A nucleotide substitution model GTR + I + G and a strict clock were used for evolution analysis.

For the complete *S* gene sequences of HCoV-229E dataset, the results of evolutionary rate (6 × 10^−4^ substitutions/site/year) and TMRCA date estimation (1950s) were approximate under four model combinations, and all ESS values exceeded 200. Besides, the average substitution rate and divergence date of HCoV-229E were in accordance with the results of the temporal signal analysis. Time-scaled Bayesian MCC tree of HCoV-229E under the model of strict clock with constant-size is shown in Figure 2. In the MCC tree, 8 obtained HCoV-229E strains were all grouped into genogroup 6 and were close to strains circulating in Japan and the United States. Furthermore, during recent years, genogroup 6 seemed to be predominant among genogroups 1 to 6.

**Figure 3:**
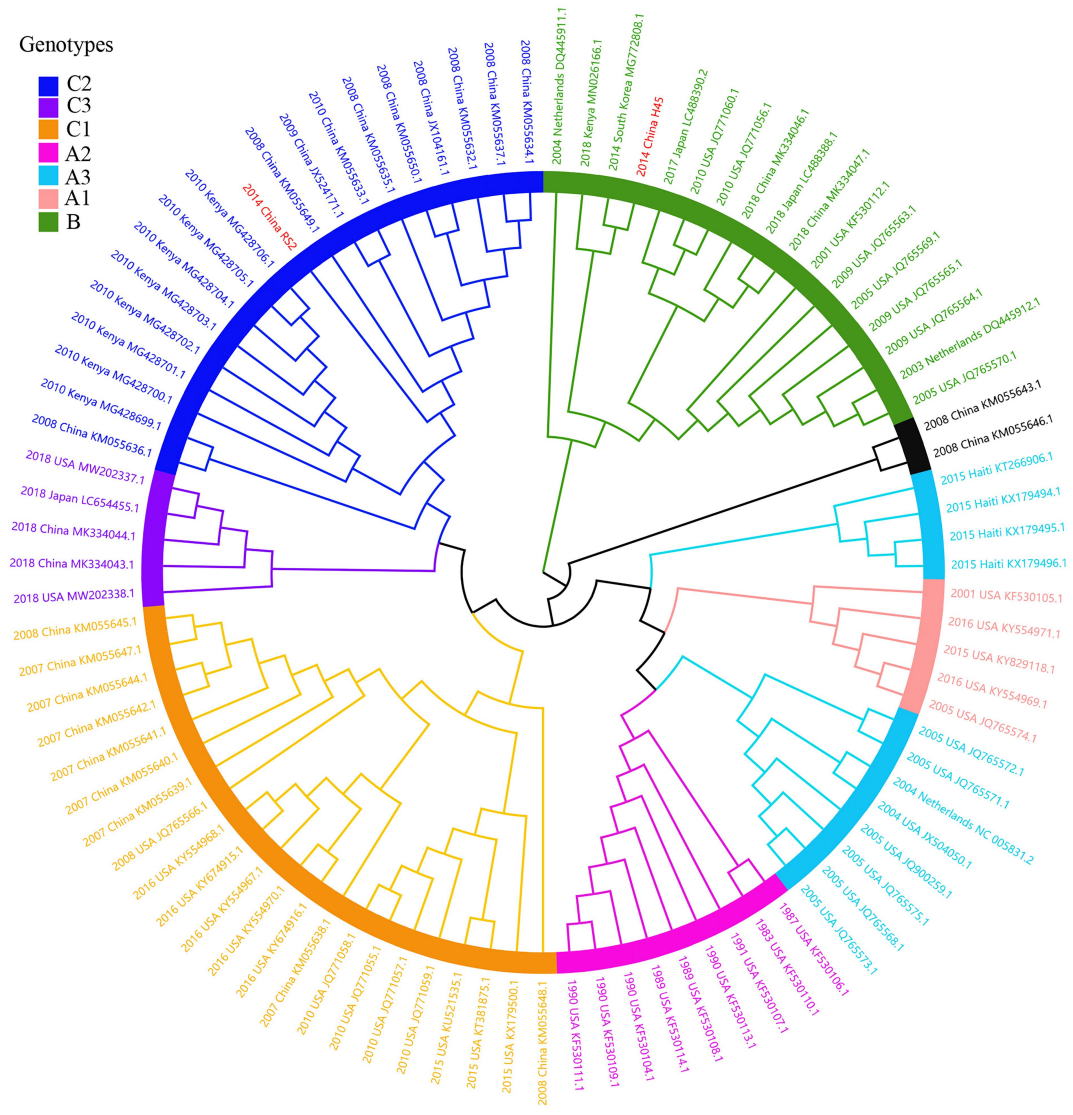
ML tree for the complete S gene of HCoV-NL63. Genotype classification is color-coded and sequences obtained in this study are colored red. The year of sampling, city and accession number are at the tip labels. Phylogenetic analysis using MEGA v6.0 with 1,000 bootstrap replicates.

**Figure 4:**
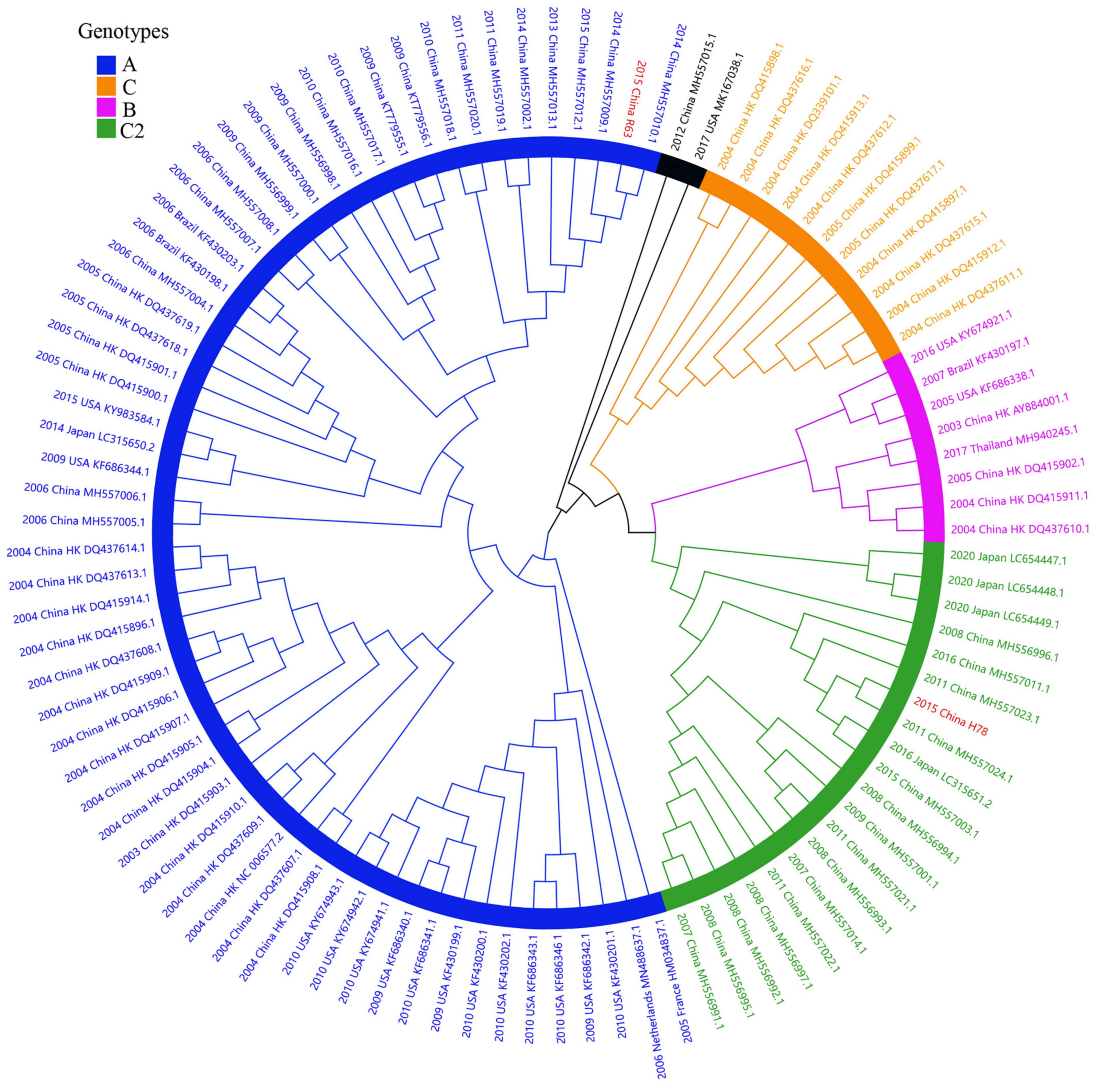
ML tree for the complete S gene of HCoV-HKU1. Different genotypes are distinguished by different colors and sequences obtained in this study are colored red. The year of sampling, city and accession number are at the tip labels. Phylogenetic analysis using MEGA v6.0 with 1,000 bootstrap replicates.

**Figure 5:**
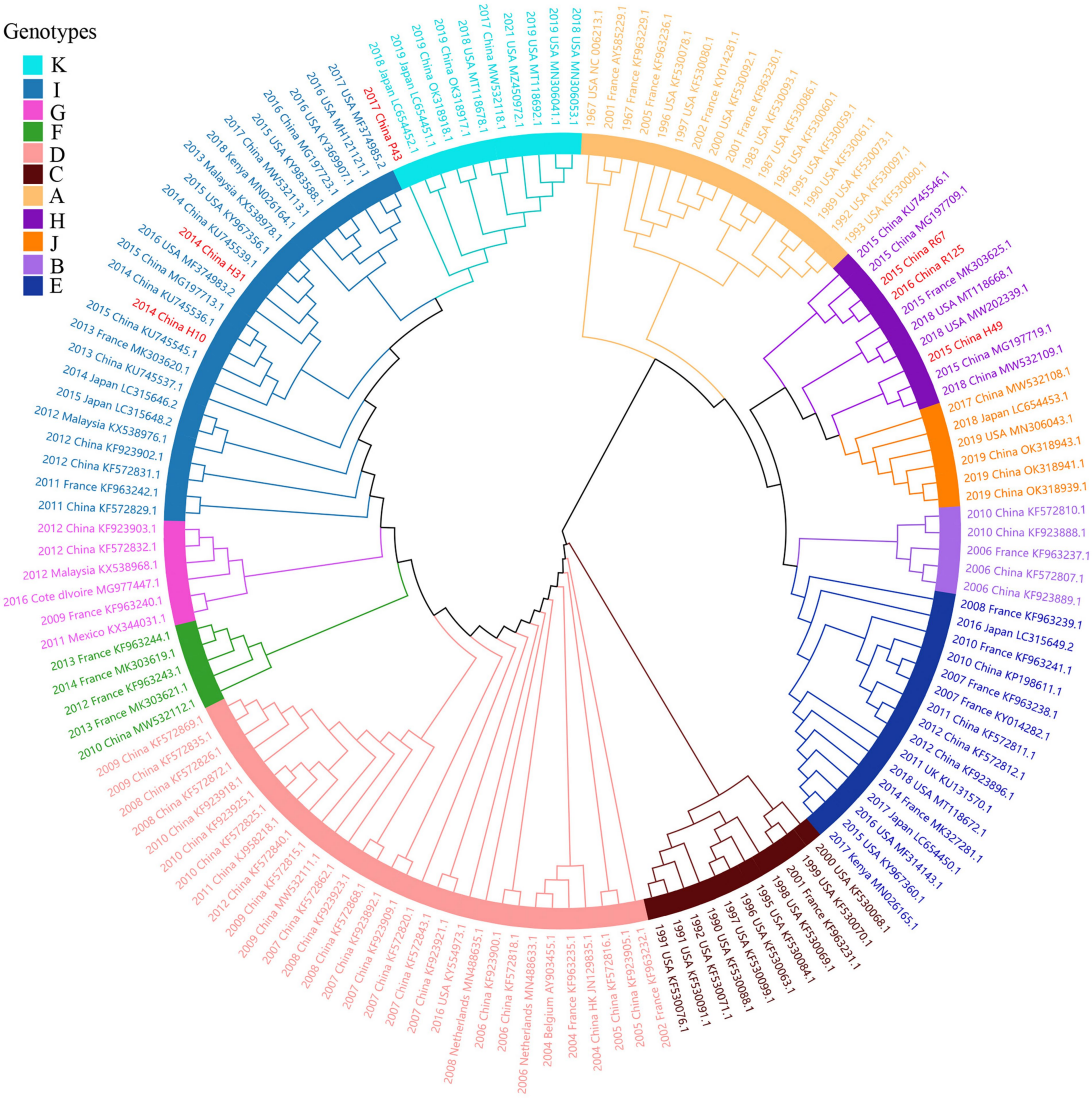
ML tree for the complete S gene of HCoV-OC43. Different genotypes are distinguished by different colors and sequences obtained in this study are colored red. The year of sampling, city and accession number are at the tip labels. Phylogenetic analysis using MEGA v6.0 with 1,000 bootstrap replicates.

Considering lower values of R^2^ and correlation coefficients of the complete *S* gene sequences of HCoV-NL63, HCoV-HKU1 and HCoV-OC43 datasets, we constructed ML tree using MEGA v6.0 to conduct phylogenetic analysis. ML trees of HCoV-NL63, HCoV-HKU1 and HCoV-OC43 are shown in Figures 3–5. The phylogenetic analysis classified the two obtained HCoV-NL63 into different genotypes, where H45 belonged to genotype B and RS2 belonged to sub-genotype C2. One HCoV-HKU1 (R63) was divided into genotype A. To clarify the division of genotype for another HKU1 (H78), we compared the evolutionary divergence among different groups. The mean distance and standard error between genotype C and the group of H78 were 0.004 ± 0.002, between genotype B and the group of H78 were 0.003 ± 0.002, and between genotype B and genotype C were 0.003 ± 0.002. By contrast, the values of other groups were all beyond 0.010. Therefore, here, we designated the generated H78 as a new sub-genotype C2 of HCoV-HKU1. Among the 6 HCoV-OC43 strains, R67, R125 and H49 were grouped in genotype H, whereas H10 and H31 were grouped in genotype I. P43 belonged to the recently reported novel genotype K. Taken together, we found that HCoV-NL63, HCoV-HKU1 and HCoV-OC43 tended to circulate in China with different coexisting genotypes, whereas HCoV-229E tended to spread with a single genotype.

### Recombination analysis

**Figure 6:**
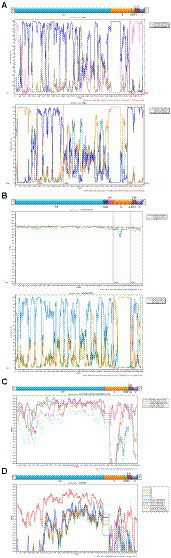
Recombination analyses of four HCoVs. Similarity plot and bootscan analysis were performed intraspecies (**A**: HCoV-NL63 and **B**: HCoV-OC43) and interspecies (**C**: HCoV-229E and BtKY229E; **D**: HCoV-NL63, HCoV-229E, BtKY229E and BtKYNL63).

The obtained 18 consensus sequences were introduced as queries in the subsequent recombination analysis. Recombination analysis was first performed within HCoV-NL63, HCoV-229E, HCoV-OC43 and HCoV-HKU1, separately. However, we detected potential recombination regions were present only in the obtained HCoV-NL63 and HCoV-OC43. In the identified HCoV-NL63 (H45 and RS2), potential recombination regions were observed in *1ab* and spanned from *S* to *N*. Among the six identified HCoV-OC43, potential recombination events were discovered in *HE* and *S* of H49, R67 and R125. Further intra-genus comparisons were performed between HCoV-NL63 and HCoV-229E, and between HCoV-HKU1 and HCoV-OC43. However, no recombination signal was detected. In order to further explore whether a recombination event occurred in the obtained HCoV-229E, and concomitantly considering the potential of virus spillover, sequences of 229E-related bat CoVs (KY073747, KY073748, and KT253269–72) were included to conduct an in-depth analysis. Unexpectedly, recombination was detected between HCoV-229E and BtKY229E. Therefore, sequences of NL63-related bat CoVs (KY073744, KY073745 and KY073746) were included in the analysis between the obtained HCoV-NL63 and HCoV-229E. Strikingly, potential recombination events were observed among HCoV-NL63, HCoV-229E, BtKY229E and BtKYNL63. Plots of recombination analysis are depicted in Figure 6.

### N-glycosylation site analysis

For the two identified HCoV-NL63 (RS2 and H45), 33 and 31 potential N-linked glycosylation sites were predicted, respectively. Compared with other strains belonging to genotype B, H45 lacked an N-glycosylation site at the aa position 511, which could be due to a base deletion. The N-glycosylation site analysis showed that the 8 HCoV-229E sequences have 27 identical potential N-glycosylation sites in the S protein, and these results were consistent with other published sequences. The predicted number of N-linked glycosylation sites in HCoV-HKU1 was 21 (H78) and 17 (R63). Among the six HCoV-OC43 sequences, H10 and H31 of genotype I shared 16 potential N-glycosylation sites with those of P43 of genotype K, whereas H49 and R67, and R125 of genotype H had 14 and 13 N-glycosylation sites, respectively. The absence of an N-glycosylation site at aa residue 648 in R125 could be due to base deletion.

### Amino acid site analysis

**Figure 7:**
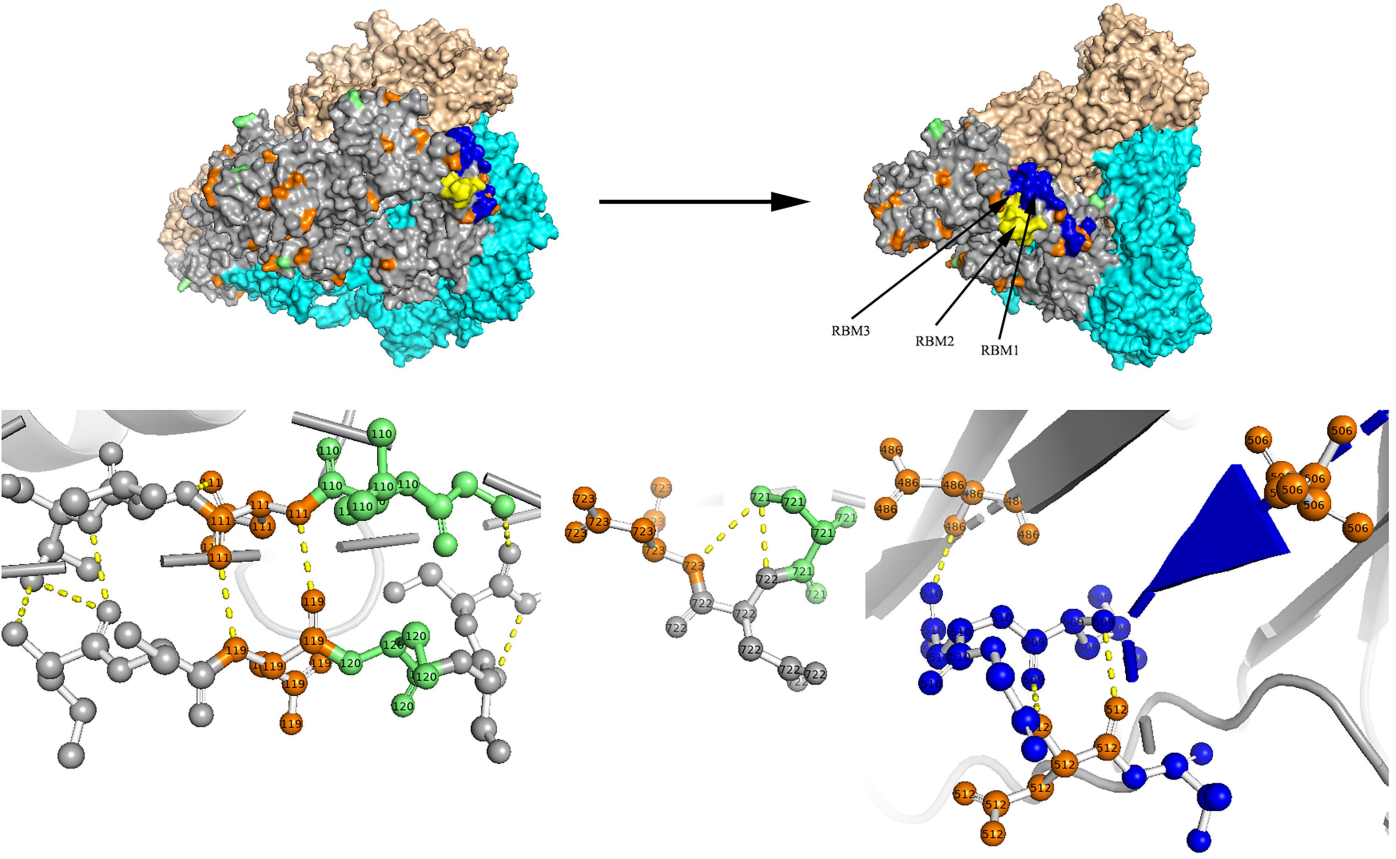
N-glycosylation and aa substitution sites of S protein of RS2. Homology modeling was using the template of 7KIP. The trimeric structure model is shown as surface and chains A to C (colored gray, cyan, and wheat, respectively). The N-glycosylation sites and amino acid substitution sites are shown in orange and green, respectively. RBM1, RBM2 and RBM3 in the RBD region are shown in blue, yellow and magenta, respectively. The dashed yellow line indicates polar contact between the residues.

**Figure 8:**
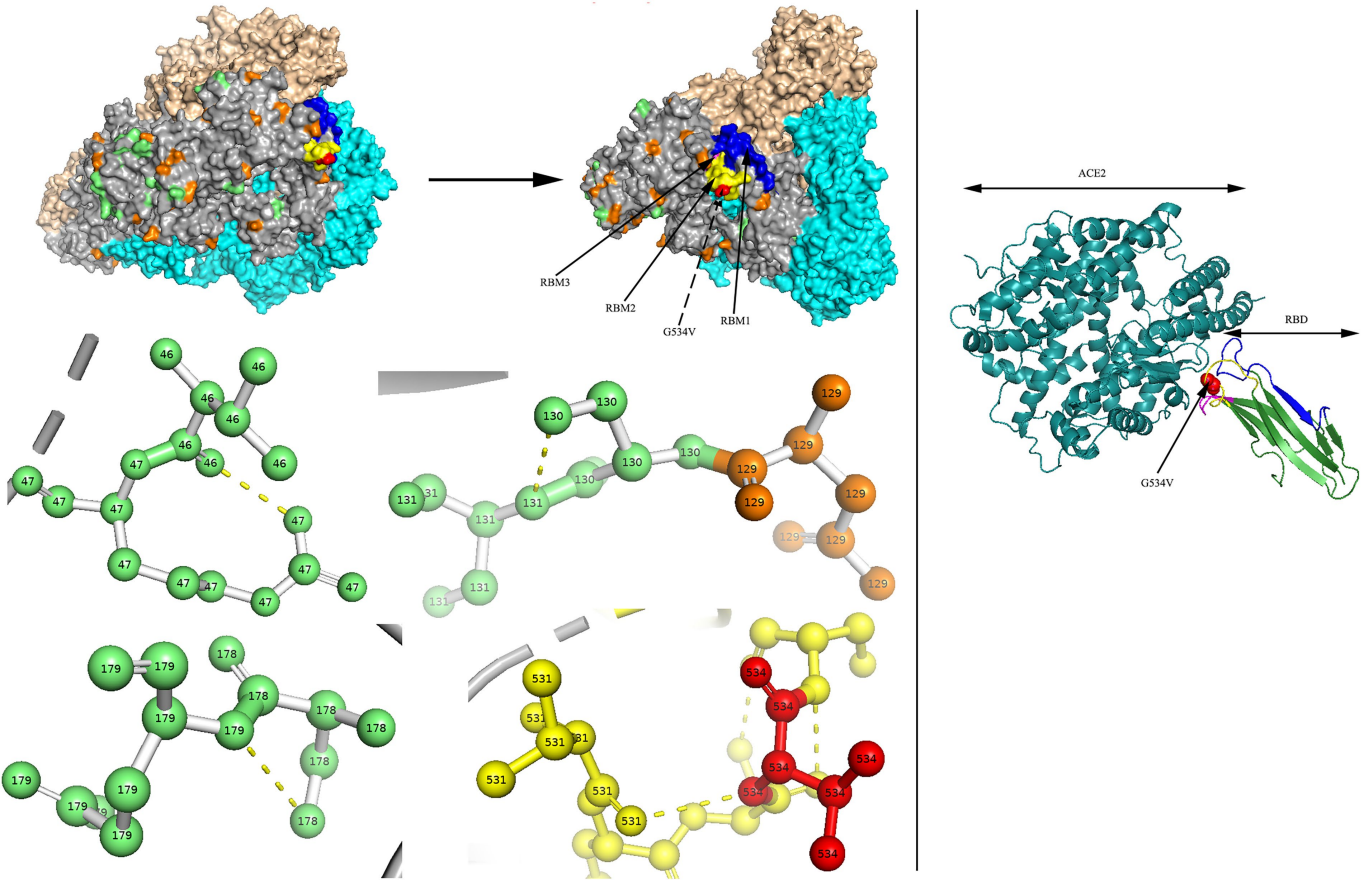
N-glycosylation and aa substitution sites of S protein of H45. Homology modeling was using the template of 7KIP. The trimeric structure model is shown as surface and chains A to C, (colored gray, cyan, and wheat, respectively). The N-glycosylation sites and amino acid substitution sites are shown in orange and green, respectively, while residue G534 is colored red. RBM1, RBM2 and RBM3 in the RBD region are shown in blue, yellow and magenta, respectively. The dashed yellow line indicates polar contact between the residues. The crystal structure of 3KBH is shown on the right.

Two models of the S protein of the two identified HCoV-NL63 sequences (RS2 and H45) were generated using the selected template of 7KIP (PDB ID). The positions of aa were defined according to reference sequence NC_005831. No site was found to be both a glycosylation site and aa substitution site. In the three-dimensional structure of RS2, the N-linked glycosylation sites at aa positions N111 and N119 made direct polar contacts, and they were linked to aa substitution sites R110I and A120S, respectively (Figure 7). Another polar contact at N-glycosylation site N723 and aa substitution site G721S. In the receptor-binding domain (RBD) region, there were two N-linked glycosylation sites (N506 and N512), of which, N512 had polar contacts with residue L509. Besides, one N-glycosylation site (N486) had a polar contact with residue N510 in the RBD region. In the three-dimensional structure of H45, three adjacent aa substitution sites T46V and H47R, L130S and L131S, and N178S and Y179E have polar contacts (Figure 8). In the receptor-binding motif (RBM2) of the RBD region, residue G534, which was considered to be critical for the binding between RBD and human ACE2, was changed from a polar glycine to a hydrophobic valine (G534V). Besides, residue V534 had a polar contact with residue V531. In order to directly display the aa substitution site G534V on the three-dimensional structure of the receptor-binding domain of HCoV-NL63, we directly labelled it on the crystal structure of 3KBH (PDB ID).

Sequence conservation of the S glycoproteins of Coronavirus is depicted in Supplementary Figure 1. Residue positions were defined according to SARS-CoV-2 (accession number: QHD43416). Apparently, conserved residues were more common in the S2 subunit, especially in the heptad repeat 1 (HR1) region. Conversely, the RBD region of the S1 subunit was considered to be hypervariable. Furthermore, in the S glycoprotein of HCoV-HKU1 belonging to genotype A, we found an aa substitution site (H512R) at residue H512 in the C-terminal domain (CTD) of the S1 subunit in R63, which occurred between two alkaline amino acids. The locations of residue were defined according to reference sequence NC_00657.

## Discussion

In this study, we obtained 18 genome sequences of four HCoVs and described their molecular features, including nucleotide identity, temporal signal, phylogenetic analysis, potential recombination event, N-glycosylation site, and major aa substitution site.

We observed a relatively high nucleotide identity within the 8 obtained HCoV-229E sequences, which might supported the clustering of the 8 HCoV-229E sequences into the same genogroup 6. For the HCoV-229E dataset, the evolutionary rate and TMRCA date estimation were similar to previous studies (Al-Khannaq et al., 2016; Su et al., 2016; Lau et al., 2021). Hitherto, HCoV-NL63, HCoV-229E, HCoV-HKU1, and HCoV-OC43 have been classified into genotypes A (A1, A2, A3) to C (C1, C2, C3), genogroups 1 to 6, genotypes A to C and genotypes A to K, respectively. In this study, no new genotype or sub-genotype were found in the obtained HCoV-229E and HCoV-NL63 sequences. For the obtained HCoV-HKU1 sequences, we named the generated H78 as a new sub-genotype C2 based on the mean distance and standard error among groups (Goya et al., 2016). Besides, we found another obtained sequence of HCoV-OC43, P43, was close to the novel genotype K circulated in Guangzhou in China (Zhang et al., 2022), denoting that the genotype shift had also occurred in the north of China. Notably, genotype shift can promote the adaptation of HCoVs in the external environment and maintain the epidemic status in the human population.

Overall, the recombination signal of each HCoV possessed individual characteristics. Interestingly, we observed that the recombination events occurred both intraspecies (in same species) and interspecies (between species). In this study, we found the two obtained HCoV-NL63 and three obtained HCoV-OC43 sequences underwent homologous recombination, while the recombination signal of obtained HCoV-HKU1 was considered to be caused by an evolutionary process other than recombination. No recombination signal was detected within the 8 obtained HCoV-229E sequences. To date, just one study has reported the detection of recombination event within HCoV-229E (Pollett et al., 2021). Recombination signal was not detected between the eight HCoV-229E and two HCoV-NL63 sequences until BtKY229E or BtKYNL63 were added. Besides, the feature of recombination signal was comparable to those of previous studies (Tao et al., 2017).

Considering the four HCoVs threaten human health continuously and the importance of N-glycosylation of *S* gene for the β-CoV (SARS-CoV-2) have been reported. We therefore made predictions to explore the characteristics of potential N-glycosylation site of *S* gene for the four HCoVs. Overall, we observed that the obtained HCoVs in the same genotype share the same N-glycosylation sites. Besides, we found the potential N-glycosylation sites of the obtained HCoVs in our study were similar to the published sequences.

The S protein of HCoVs is composed of the S1 and S2 functional subunits, corresponding to the receptor-binding and membrane-fusion regions, respectively (Xia et al., 2019). In HCoV-NL63, three receptor-binding motifs (RBM1, RBM2 and RBM3) in the RBD region have been considered to directly contact ACE2 (Wu et al., 2009). In HCoV-HKU1, a previous study had suggested that five residues (V509, L510, D511, H512, and W515) in the CTD are essential for binding to neutralizing antibodies (Ou et al., 2017). In this study, we identified two aa substitution sites at residues G534 and H512 in the RBM2 and CTD regions, respectively. Additionally, the S2 subunit was more conserved than the S1 subunit for the 18 obtained HCoVs, which resembled the highly contagious viruses (SARS-CoV and SARS-CoV-2). Furthermore, below the aligned sequences, residues labelled with “e” and “g” in heptad repeat 1 (HR1) and “a” and “d” in heptad repeat 2 (HR2) have been reported to have interactions (Supplementary Figure 1; Xia et al., 2019). The above aa substitution sites and residue conversation analyses of the S glycoprotein may be conducive to provide therapeutic and prophylactic clues.

In conclusion, we analysed 18 obtained genome sequences in the study, including 2 HCoV-NL63, 8 HCoV-229E, 2 HCoV-HKU1 and 6 HCoV-OC43. To characterize the four HCoVs and provide better insights, on one hand, we performed an in-depth analysis involving nucleotide identity and potential recombination event according to the whole genome; on the other hand, we clarified the molecular evolution and important residues based on the full-length sequence of the *S* gene. These findings could serve as a clue for exploring virus emergence, epidemic, transmission and evolution, which would ultimately help the early diagnosis, surveillance, treatment, and prevention of coronavirus infections.

## Data availability statement

The GenBank accession numbers are OK073075-OK073092 and the accession number in NCBI is PRJNA 843130 (SAMN28704067–SAMN28704084). Figshare (https:// doi.org/10.6084/m9.figshare.20521257.v1).

## Ethics statement

The studies involving human participants were reviewed and approved by Medical Ethics Committee of Beijing Children’s Hospital, Capital Medical University. Written informed consent to participate in this study was provided by the participants’ legal guardian/next of kin.

## Author contributions

TZ, FY, and NS designed the project and analyzed the data. CZ, XC, SA, ZX, and BX collected the samples. NS, TZ, JD, and LS conducted the experiments. NS wrote the manuscript. All authors contributed to the article and approved the submitted version.

## Funding

This work was supported by the National Key Research and Development Program of China 2021YFC2302003 and the CAMS Innovation Fund for Medical Sciences (grant no.2021-I2M-1-039).

## Conflict of interest

The authors declare that the research was conducted in the absence of any commercial or financial relationships that could be construed as a potential conflict of interest.

## Publisher’s note

All claims expressed in this article are solely those of the authors and do not necessarily represent those of their affiliated organizations, or those of the publisher, the editors and the reviewers. Any product that may be evaluated in this article, or claim that may be made by its manufacturer, is not guaranteed or endorsed by the publisher.

## Footnotes

^1^ https://www.geneious.com

^2^ https://www.ebi.ac.uk/services

^3^ http://beast.community/

^4^ https://sray.med.som.jhmi.edu/SCRoftware/
