# Supplementary figures and images for: Molecular evolution of human coronavirus-NL63, -229E, -HKU1 and -OC43 in hospitalized children in China
Source: Front Microbiol. 2022 Nov 2;13:1023847. doi: 10.3389/fmicb.2022.1023847 (PMC9666422; doi:10.3389/fmicb.2022.1023847)

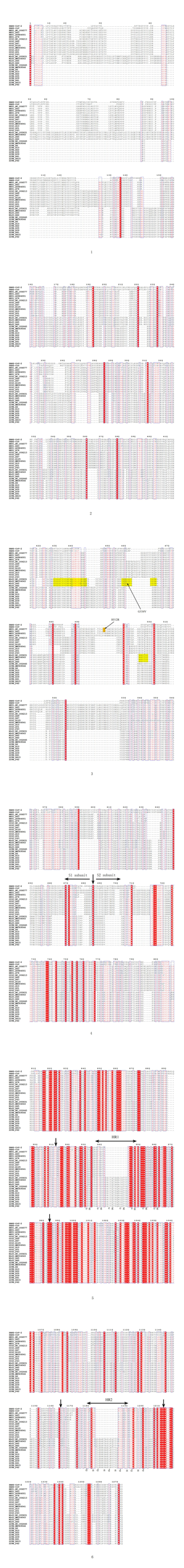

Supplement: Supplementary file 1 [file Image_1.JPEG]
