# Supplementary material for: Molecular evolution of human coronavirus-NL63, -229E, -HKU1 and -OC43 in hospitalized children in China
Source: Front Microbiol. 2022 Nov 2;13:1023847. doi: 10.3389/fmicb.2022.1023847 (PMC9666422; doi:10.3389/fmicb.2022.1023847)
Supplement: Supplementary file 2 [file Table_1.DOC]

Table 1 Information of four human coronaviruses identified in this study

| Virus | Accession number | Name | Year | Age | Gender | Sample | City | Genome |
| --- | --- | --- | --- | --- | --- | --- | --- | --- |
| NL63 | OK073075 | H45 | 2014 | 0.98 | female | Nasopharyngeal aspirates | Hebei | Gap in 1ab (234bp) and S (72bp) |
| NL63 | OK073076 | RS2 | 2017 | 1.00 | female | Nasopharyngeal aspirates | Beijing | Complete |
| 229E | OK073077 | R2 | 2018 | 4.00 | female | Nasopharyngeal aspirates | Beijing | Complete |
| 229E | OK073078 | R10 | 2017 | 3.00 | male | Nasopharyngeal aspirates | Beijing | Nearly Complete |
| 229E | OK073079 | R22 | 2018 | 0.05 | male | Nasopharyngeal aspirates | Beijing | Complete |
| 229E | OK073080 | R35 | 2018 | 2.00 | male | Nasopharyngeal aspirates | Beijing | Nearly Complete |
| 229E | OK073081 | R39 | 2018 | 0.05 | female | Nasopharyngeal aspirates | Beijing | Nearly Complete |
| 229E | OK073082 | R41 | 2018 | 5.00 | male | Nasopharyngeal aspirates | Beijing | Complete |
| 229E | OK073083 | RS15 | 2017 | 0.72 | male | Nasopharyngeal aspirates | Beijing | Complete |
| 229E | OK073084 | P42 | 2017 | 0.50 | male | Nasopharyngeal aspirates | Beijing | Complete |
| HKU1 | OK073085 | R63 | 2015 | 6.00 | female | Nasopharyngeal aspirates | Beijing | Complete |
| HKU1 | OK073086 | H78 | 2015 | 3.00 | male | Nasopharyngeal aspirates | Hebei | Complete |
| OC43 | OK073087 | H10 | 2014 | 0.28 | male | Nasopharyngeal aspirates | Hebei | Complete |
| OC43 | OK073088 | H31 | 2014 | 0.50 | female | Nasopharyngeal aspirates | Hebei | Gap in 1ab (5bp) |
| OC43 | OK073089 | H49 | 2015 | 0.09 | female | Nasopharyngeal aspirates | Hebei | Complete |
| OC43 | OK073090 | P43 | 2017 | 0.92 | male | Nasopharyngeal aspirates | Beijing | Complete |
| OC43 | OK073091 | R67 | 2015 | 2.00 | male | Nasopharyngeal aspirates | Beijing | Complete |
| OC43 | OK073092 | R125 | 2016 | 0.83 | male | Nasopharyngeal aspirates | Beijing | Gap in 1ab (100 bp) and S (24bp) |
