# Supplementary material for: Molecular evolution of human coronavirus-NL63, -229E, -HKU1 and -OC43 in hospitalized children in China
Source: Front Microbiol. 2022 Nov 2;13:1023847. doi: 10.3389/fmicb.2022.1023847 (PMC9666422; doi:10.3389/fmicb.2022.1023847)
Supplement: Supplementary file 3 [file Table_2.DOC]

| Virus | Nucleotide identity (%) | | | | | |
| --- | --- | --- | --- | --- | --- | --- |
|  | CDS | 1ab | S | E | M | N |
| Alphacoronavirus |  |  |  |  |  |  |
| HCoV-NL63 | 95.8-99.8  (96.2) | 95.4-99.8  (95.8) | 95.4-99.8  (96.2) | 95.4-100  (98.3) | 95.4-100  (98.5) | 95.4-100  (99.0) |
| HCoV-229E | 98.1-99.9  (99.7-100) | 98.4-99.9  (99.7-100) | 96.1-99.9  (99.8-100) | 99.1-100  (100) | 97.9-100  (99.4-100) | 97.4-100  (99.4-100) |
| Betacoronavirus |  |  |  |  |  |  |
| HCoV-HKU1 | 93.3-99.3  (93.5) | 95.8-99.2  (96.0) | 84.2-99.8  (84.1) | 85.9-99.6  (85.9) | 92.0-100  (92.1) | 93.3-100  (93.4) |
| HCoV-OC43 | 97.3-100  (97.9-99.8) | 97.5-100  (98.4-99.8) | 94.4-100  (94.6-99.8) | 89.7-100  (90.5-100) | 94.8-100  (99.0-100) | 91.1-100  (98.5-100) |

Table 2 Nucleotide identity of four human coronaviruses
